# Remote data collection to detect asthma exacerbations: A decentralized approach to clinical research in asthma

**DOI:** 10.1017/cts.2026.10697

**Published:** 2026-02-10

**Authors:** Allison J. Burbank, Jeremy Owens, Andre Espaillat, Claire E. Atkinson, Stephen A. Schworer, Katherine M. Whaylen, Kelly Chason, Michelle L. Hernandez

**Affiliations:** 1 Pediatrics, The University of North Carolina at Chapel Hill School of Medicine, USA; 2 Tidewater Allergy & Asthma, USA; 3 Oklahoma Allergy & Asthma, USA; 4 Medicine, The University of North Carolina at Chapel Hill School of Medicine, USA; 5 Pediatrics, The University of North Carolina at Chapel Hill, USA

**Keywords:** Asthma, adolescents, digital health, remote data capture, decentralized clinical research

## Abstract

**Background:**

Decentralized trial designs can improve accessibility and continuity of research participation by enabling remote data collection. This manuscript describes our team’s experiences with remote data collection to identify acute asthma exacerbations in a clinical study as well as practical insights that support the continued optimization of remote methodologies.

**Methods:**

In this 12-month observational study, adolescents aged 12–21 years with persistent asthma and ≥1 exacerbation in the prior 24 months completed an initial in-person visit followed by monthly virtual visits. Participants used home spirometry, app-based symptom tracking, smart inhalers to monitor lung function and short-acting beta agonist (SABA) use, and self-collection of nasal epithelial lining fluid (NELF) samples. Exacerbations were defined *a priori* by symptom/SABA thresholds or ≥20% FEV_1_ decline.

**Results:**

Forty participants enrolled; 73% completed all visits. Median adherence to performance of daily spirometry and symptom surveys was 44% and 38%, respectively. Seventy-eight percent experienced ≥1 exacerbation. Of 132 alerts, 80% represented true exacerbations, primarily due to ≥20% FEV_1_ decline; erroneous alerts were linked to software errors and poor spirometry technique. Sixty-six NELF sample sets were collected and 50 were analyzed. Cytokine concentrations did not differ significantly between clinic-collected and self-collected samples. Technical challenges included device connectivity issues, erroneous alerts, and shipping delays.

**Conclusions:**

Decentralized study designs with remote data collection requires further study as a means of conducting clinical research in asthma that increases participant accessibility, representation and generalizability of trial results. This approach presents numerous challenges and requires further optimization to address adherence, technical complexity, and staff burden while maintaining scientific rigor.

## Introduction

Asthma is among the most common chronic diseases in the U.S, affecting approximately 9% of U.S. adolescents [1]. Asthma is characterized by variable airflow obstruction that occurs after exposure to triggers, resulting in asthma “attacks,” or exacerbations, that over time contribute to airway remodeling and permanent loss of lung function [2,3]. This variability in symptoms and objective findings presents challenges for clinical care and asthma-focused clinical research, since data collection typically occurs at discrete clinic or study “visits” that often correspond with periods of low asthma symptoms and reduced or absent airway obstruction. This “snapshot” of data may poorly represent the patient’s overall impairment from asthma and risk of future exacerbations, which may lead providers and investigators to overestimate asthma control and under-treat the disease. This is particularly problematic in children and adolescents with asthma who often have low day-to-day impairment despite elevated risk of asthma exacerbations [4]. Adolescents are particularly vulnerable to poor health outcomes related to asthma and experience higher rates of asthma-related death than younger children [5,6]. Adolescents are more likely to normalize and under-report asthma symptoms, with poor recognition of deteriorating asthma control and delays in seeking care [7–9].

With an estimated 95% of adolescents having access to a smartphone [10], and 65% of young adults using apps to track their health [11], digital health devices and software platforms offer potential solutions to the obstacles of poor disease perception and the limitations in subjective and objective data availability in adolescent asthma management and clinical research. Compact home spirometers, smartphone apps, and digital inhalers capture lung function measurements and inhaled medication use, respectively, while minimizing the need for user data entry. This technology has shown promise for improving Asthma Control Test (ACT®) scores, increasing rescue medication-free days [12,13], and predicting asthma exacerbation events in adults with obstructive lung disease [14]. Methods to accurately measure and track subjective and objective indicators of loss of asthma control in adolescents are needed to (1) allow for earlier intervention that can reduce the risk of progression to severe symptoms, and (2) improve the feasibility, quantity and quality of data collection in clinical research.

The SARS-CoV-2 pandemic placed restrictions on in-person research activities, including the ability to perform aerosol-generating procedures such as spirometry. Changes made in response to the challenges faced during the pandemic demonstrated the feasibility of remote visits and data collection in research. Remote data collection methods support the decentralization of clinical research studies, making research participation more feasible for families with working parents, those with limited means of transportation, children and adolescents attending school, and individuals living in rural communities who would otherwise need to travel long distances to participate.

This manuscript describes our team’s experiences – highlighting both successes and challenges – with near-fully remote data collection in a clinical research study of asthma in adolescents. While the feasibility of remote data collection was not the primary intent of the study, the shift to remote study activities during the pandemic presented an opportunity to challenge traditional methods of data collection in clinical research. We offer practical insights that support the continued innovation and optimization of remote methodologies, enabling clinical research that is patient centered.

The purpose of this observational study was to measure changes in nasal airway inflammatory cytokine production during an acute asthma exacerbation in adolescents and young adults (age 12–21 years) with persistent asthma. The interleukin-1 cytokine family (IL-1α and IL-1β) is a key mediator in many inflammatory disease states, including asthma. IL-1 Receptor Antagonist (IL-1RA) is an endogenous antagonist of the IL-1α and IL-1β receptors that regulates the activity of these inflammatory cytokines. We hypothesized that IL-1 is a key determinant of asthma exacerbation frequency and severity. In response to the SARS-CoV-2 pandemic, the study was designed to minimize in-person visits, to allow for remote collection of symptoms, inhaler usage, and lung function measurement, and to incorporate self-collection of biological samples by study participants.

## Materials and methods

### Study population

Adolescents and young adults aged 12 to 21 years with physician-diagnosed persistent asthma were recruited from University of North Carolina pediatric allergy and pulmonology clinics. Adolescents were eligible to participate if they were currently using an asthma controller medication such as an inhaled corticosteroid (ICS) or ICS in combination with long-acting beta agonist (LABA) or leukotriene receptor antagonist (LTRA), had a history of at least one exacerbation requiring systemic corticosteroids in the past two years, and had access to a smartphone and wireless internet in their primary residence. Use of biologic drugs for treatment of asthma was permitted. Participants with systemic corticosteroid-dependent asthma, other pulmonary disease that might affect interpretation of symptoms or spirometry (such as vocal cord dysfunction), inability to perform spirometry, or a history of spirometry-induced bronchospasm were excluded. All participants provided written informed consent or assent. The study received approval by the University of North Carolina Institutional Review Board.

### Study design

Participants completed a single in-person visit followed by monthly virtual visits over the 12-month study period. Data collected at the initial study visit included Asthma Control Test® (ACT) score, asthma-focused medical history, pre- and post-bronchodilator spirometry and fractional exhaled nitric oxide (FeNO) (NIOX®, Morrisville, NC, USA). Participants received an FDA-cleared handheld spirometer (Spirobank Smart®, MIR Inc., New Berlin, WI, USA) for home use [15]. This app-based device is commonly utilized in clinical trials and used to measure spirometry parameters as outcome measures in studies related to asthma, COPD, and interstitial pulmonary fibrosis [15–20]. The software app (VitalFloⓇ, Raleigh, NC, USA) used American Thoracic Society (ATS)/European Respiratory Society (ERS) standards to determine whether the spirometry maneuvers met acceptability, usability and reproducibility standards [21]. The participant’s smartphone displayed an indicator of whether their spirometry attempt was considered acceptable in accordance with ATS/ERS standards (a smiling face icon if acceptable technique, or a frowning face icon if unacceptable, followed by a prompt to try again). The best of three acceptable spirometry attempts was transmitted via Bluetooth from the smartphone app to a cloud-based HIPAA-compliant database where study staff regularly reviewed flow-volume loops to assess technique. If their spirometry indicated subpar technique, study staff arranged for virtual re-training sessions with participants. Spirometry values were never displayed to the participants, who were asked to use the spirometer once daily in the evening and to complete a brief asthma symptom survey in the app each night. Participants received one daily push notification to their device reminding them to the complete their spirometry and survey. In addition to incentives provided for attending study visits, participants received additional monetary incentives for each month in which they were adherent to daily spirometry and survey completion on four of seven days each week averaged over the month. A digital Hailie® Smartinhaler was installed onto the participant’s rescue inhaler to record each actuation of rescue medication during the study period and transmit this data to a cloud-based database. At each monthly virtual visit, participants were queried about any interim unscheduled visits to their primary care provider, urgent care, emergency room, or hospitalization related to their asthma or any use of systemic corticosteroids. Spirometry, symptom survey data and rescue medication use were matched to the participant-reported asthma exacerbations.

Participants were also trained in self-collection of nasal epithelial lining fluid (NELF) samples for analysis of nasal IL-1 family cytokines. NELF samples were collected during the initial in-person study visit, and participants collected a second set of NELF samples at home the day of the in-person visit (baseline) and with each asthma exacerbation (defined below). NELF was collected by inserting two small paper strips into each nostril and held in place for two minutes then removed and stored in the home’s standard freezer. Lab staff overnighted a shipping box containing the materials needed to return samples, prepaid shipping labels, a cryosafe cold box, ice packs, and USB temperature datalogger to track temperature change during return shipping.

### Asthma exacerbation events

**Figure 1.**
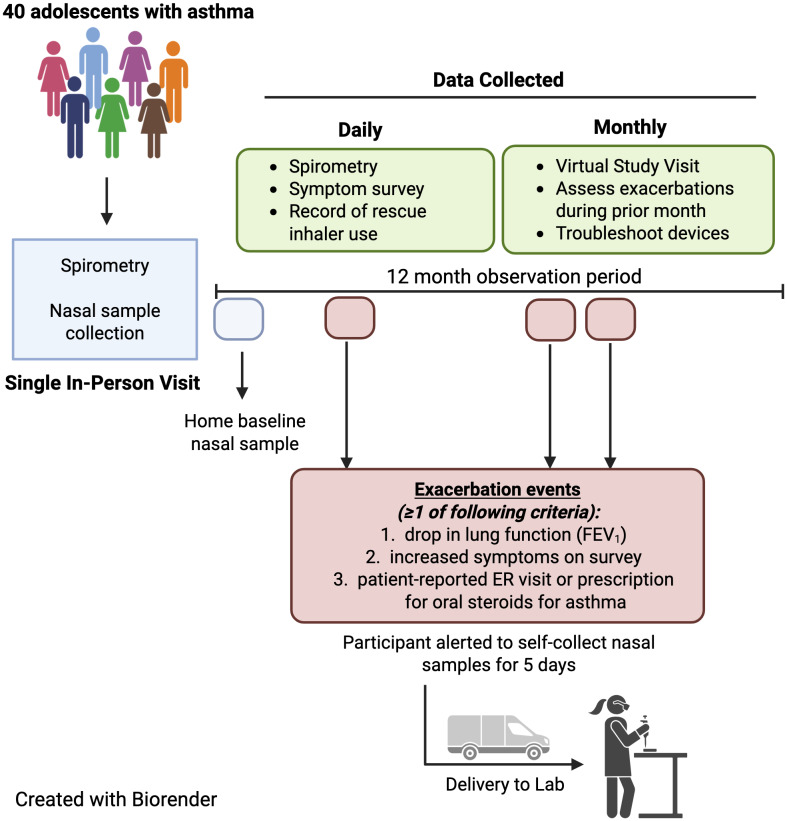
This schematic provides an overview of study workflow and participant engagement timeline.

During the 12-month study period, participants were prompted by the smartphone app to self-collect NELF samples once daily for five days if one or more of the following criteria for asthma exacerbation were met: (1) an increase in asthma symptoms and rescue medication use reported in the daily survey, defined as ≥6 puffs of albuterol in a six hour period; ≥12 or more puffs of albuterol in a 24 hour period; night awakenings leading to albuterol use for two of three consecutive nights; or use of ≥8 puffs of albuterol on two of three consecutive days [22]; or (2) reduction in FEV1 of ≥20% from their baseline measurement collected during a period of wellness (considered a clinically significant change [21]) (Figure 1). If participants were alerted to self-collect nasal samples, an alert was also sent to the study coordinator who contacted the participant to confirm that exacerbation criteria were met before sample collection was begun. Additionally, if a participant self-reported a prescription for systemic corticosteroids (oral, intramuscular, or intravenous) for asthma exacerbation, the study coordinator instructed the participant to begin collecting nasal samples for five days. If NELF samples were collected outside of the five-day window from the start of exacerbation, those samples were excluded from the final analysis. For subsequent exacerbations, NELF samples were only collected if ≥4 weeks had passed since the prior exacerbation with clear resolution of symptoms in the interim. Study staff also monitored participants’ electronic health records (EHR) to identify asthma exacerbations.

## Results

### Demographics & baseline characteristics

The study was conducted from July 2021 to January 2024. We screened 113 adolescents and enrolled 40. Demographic characteristics of the study population are shown in Table 1. The median age (interquartile range, IQR) was 15 years (13, 16), and 48% of the study population identified as female. The study sample was racially, ethnically and socioeconomically diverse, 65% non-White and 20% self-reporting annual household income below the federal poverty level. The median FEV1 percent predicted was 82% (77, 87), which is considered normal. Despite a median ACT score of 21 (19, 23), consistent with well controlled asthma, self-reported disease burden was high among the study population, with 88% of participants reporting an asthma exacerbation requiring systemic corticosteroids in the prior year. Additionally, 50% of participants reported a prior history of hospitalization due to asthma, 28% required ICU care and 5% required intubation with mechanical ventilation due to asthma exacerbation in the past.

**Table 1.** Characteristics of the study population (*N* = 40)

Demographics	
Age^a^	15 (13, 16)
Female sex^b^	19 (48%)
Race^b^	
African American	19 (47%)
Caucasian	14 (35%)
American Indian/Alaskan Native	3 (8%)
More than one race	4 (10%)
Ethnicity^b^	
Hispanic/Latine	1 (3%)
Non-Hispanic/Latine	39 (97%)
Body Mass Index^a^	24 (22, 29)
Co-morbid allergic disease^b^	
Allergic rhinitis	38 (95%)
Atopic dermatitis	23 (56%)
Food allergy	16 (40%)
Household annual pre-tax income	
Below FPL	8 (20%)
At or above FPL	29 (73%)
Chose not to answer	3 (7%)

Asthma Disease Burden	
Age at asthma diagnosis^a*^	3 (2, 6)
Exacerbations requiring OCS in prior 12 mos^b*^	
0	5 (12%)
1	20 (50%)
≥2	15 (38%)
History of ≥1 hospitalization for asthma^b*^	20 (50%)
History of ≥1 PICU admission for asthma^b*^	12 (30%)
Intubation for asthma^b*^	2 (5%)
Prescribed asthma medication at study entry^b*^	
ICS	12 (30%)
ICS/LABA	28 (70%)
LTRA	16 (40%)
LAMA	4 (10%)
Biologic	12 (30%)
Pre-bronchodilator FEV1 % predicted^a^	82 (77, 87)
Asthma Control Test (ACT) score^a^	21 (19, 23)
Secondhand smoke exposure^b*^	9 (23%)

a Median (interquartile range).

b *N* (%).

* Based on self-report or parent-report.

FPL = federal poverty limit; OCS = oral corticosteroid; ICS = inhaled corticosteroid; ICS/LABA = inhaled corticosteroid and long-acting beta agonist combination; LTRA = leukotriene receptor antagonist; LAMA = long-acting muscarinic antagonist.

### Adherence to visits and study procedures

**Figure 2.**
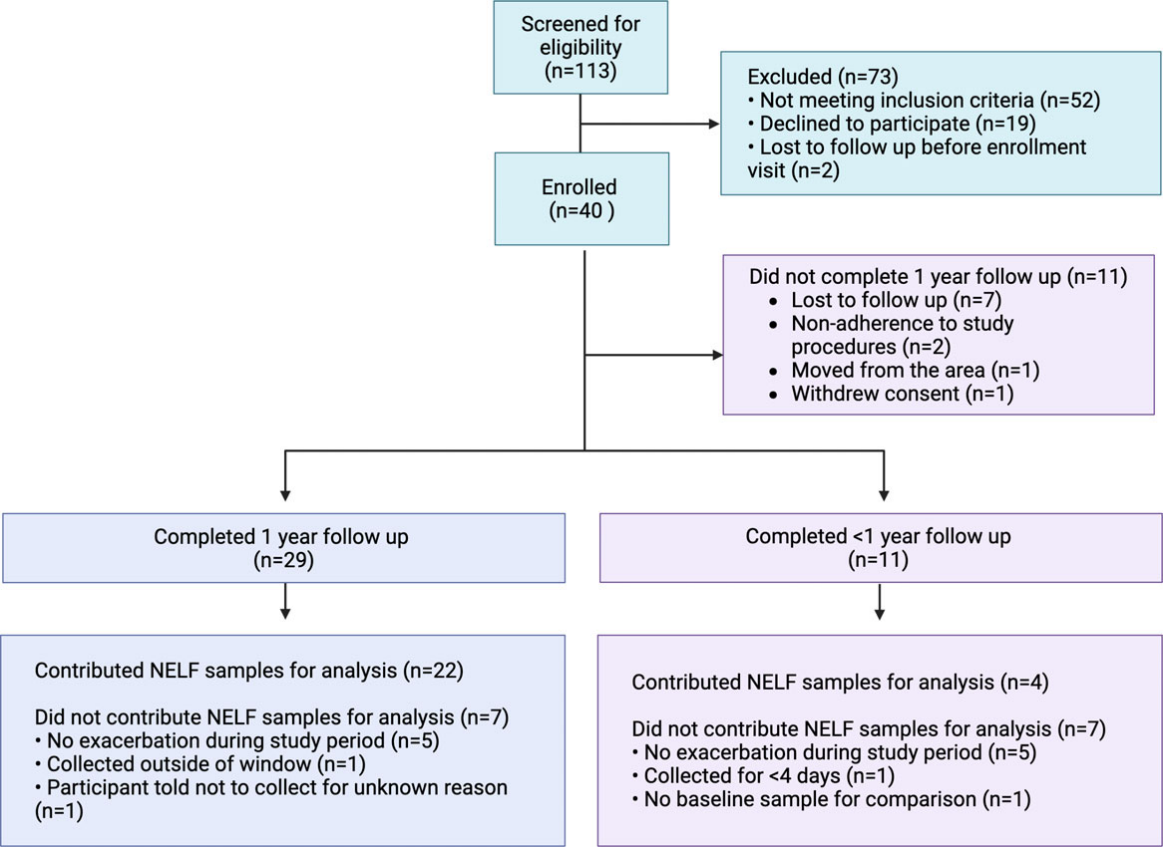
Participant flow through the study.

Twenty-nine out of 40 participants (73%) completed all 13 study visits over the 12-month study period. Reasons for study discontinuation included loss to follow up (64%), poor adherence to study procedures and visits (18%), moving out of the area (9%) and withdrawal of consent (9%) (Figure 2). Among participants who completed all study visits (*n* = 29), daily spirometry measurement was performed a median (IQR) of 44% (23, 60) of enrolled days, and daily survey completion on 38% (21, 63) of enrolled days. The group that discontinued the study early (*n* = 11) completed a median (IQR) 67% (58, 75) of study visits. Adherence to daily spirometry and survey completion was lower among those that discontinued the study early, 13% (6, 23) and 18% (11, 33) of enrolled days, respectively. We observed no significant difference in FEV1 measured at the in-person visit with coaching by research staff (median FEV1 2.68, 95% CI 2.41, 2.96) and the first FEV1 measured by the participant at home with coaching by the device software (≤7 days from the in-person measurement) (2.7, 95% CI 2.62, 2.8; *p* = 0.4 using Wilcoxon matched pairs signed rank test of paired data). The digital rescue inhalers detected a total of 4949 actuations of rescue inhaler in 37 of 40 (93%) participants. Three participants did not use the digital rescue inhaler at all during the study.

### Asthma exacerbation events

Thirty-one participants (78%) experienced ≥1 asthma exacerbations per the pre-defined criteria during the 12-month study period. A total of 23 (58%) participants experienced multiple exacerbations during the study period, eight (20%) participants experienced a single exacerbation, and nine (22%) participants experienced no exacerbations. Among participants with multiple exacerbations, the number of discrete exacerbation events ranged from two to nine episodes during the study period. An event was considered an exacerbation if it met one or more of our pre-specified criteria for symptoms (based on the daily app-based survey), lung function (≥20% decrease in FEV1 from baseline), self-reported or EHR documented use of systemic corticosteroids, ED visits or hospitalization because of asthma. For participants who experienced multiple exacerbations during the 12-month study period, an exacerbation event was considered unique if it occurred ≥4 weeks from the previous exacerbation, with clear improvement or resolution of symptoms in between the events.

**Figure 3.**
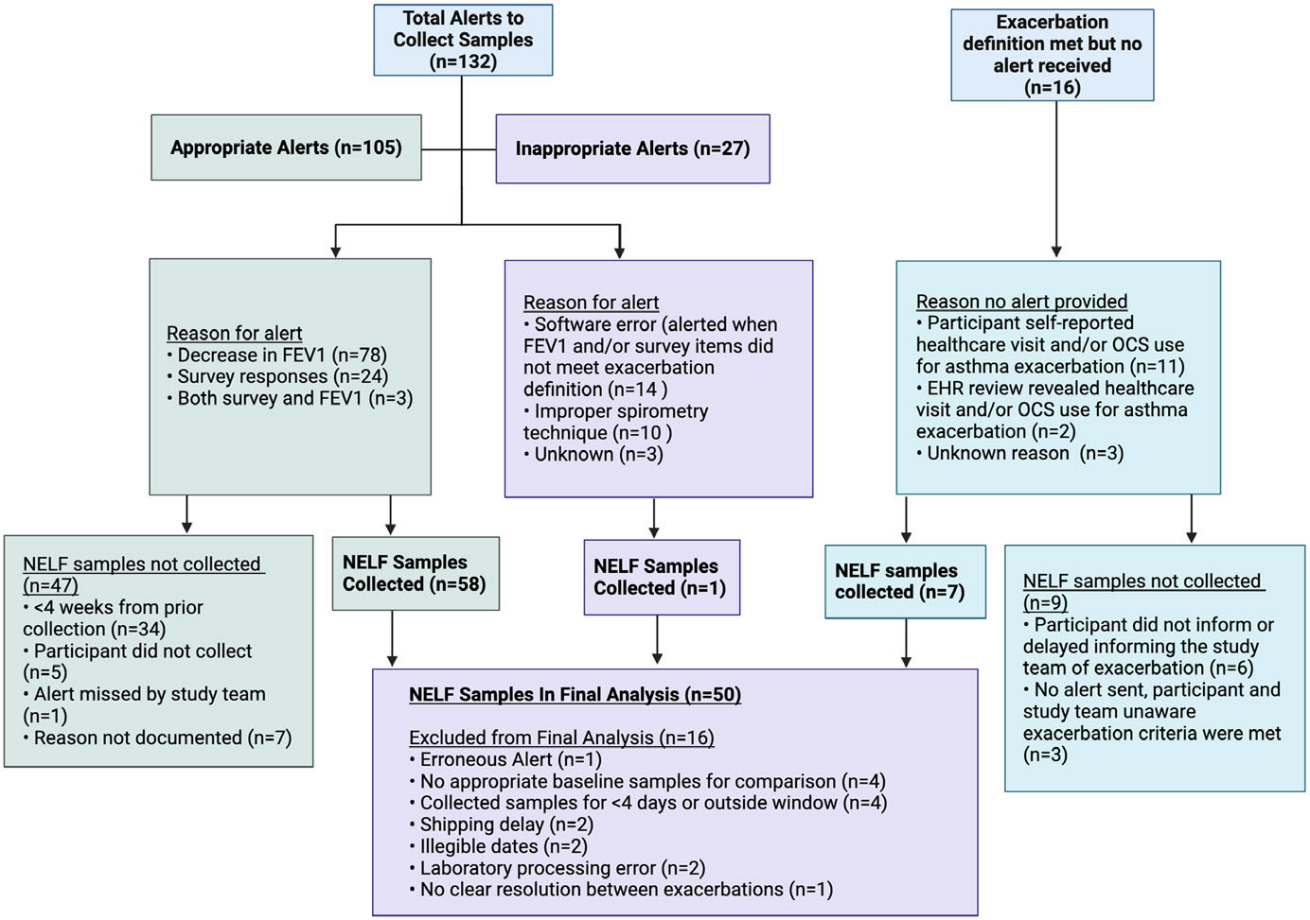
Flow chart illustrating exacerbations identified by software alerts (appropriate alerts) and by self-report or review of the electronic health records, as well as inappropriate alerts received, and corresponding NELF samples collected.

A total of 132 alerts to collect NELF samples were sent to participants indicating that the criteria for acute asthma exacerbation were met (Figure 3). Upon review by study staff, 105 (80%) of alerts truly met criteria for an exacerbation, with the most common reason being a ≥20% decrease in FEV1 (74%). NELF samples were collected in 58 (55%) of these events. The most common reason why NELF was not collected during an exacerbation event was inadequate time since the previous exacerbation (<4 weeks). The remaining 27 alerts were erroneous, the most common reasons being software errors (52%) and suspected improper spirometry technique (37%). Only one erroneous alert resulted in collection of NELF samples, which were excluded from the final analysis. There were 16 exacerbations in which no alert was received. This was mostly due to participants self-reporting healthcare visits or systemic steroid prescriptions for asthma but who were not recording spirometry or survey data around the time of the exacerbation (69%). Two exacerbations (13%) were not reported to the study team by the participant but were identified through review of their electronic health records. NELF samples were collected for 7 (44%) of these 16 “no alert received” exacerbation events, while no samples were collected from the remaining nine exacerbations. The most common reason for not collecting samples was related to significant delays (>5 days) in participants’ notifying the study team of exacerbations. In total, 66 sets of NELF samples were collected and 50 were included in the final analysis.

### Sample integrity

**Figure 4.**
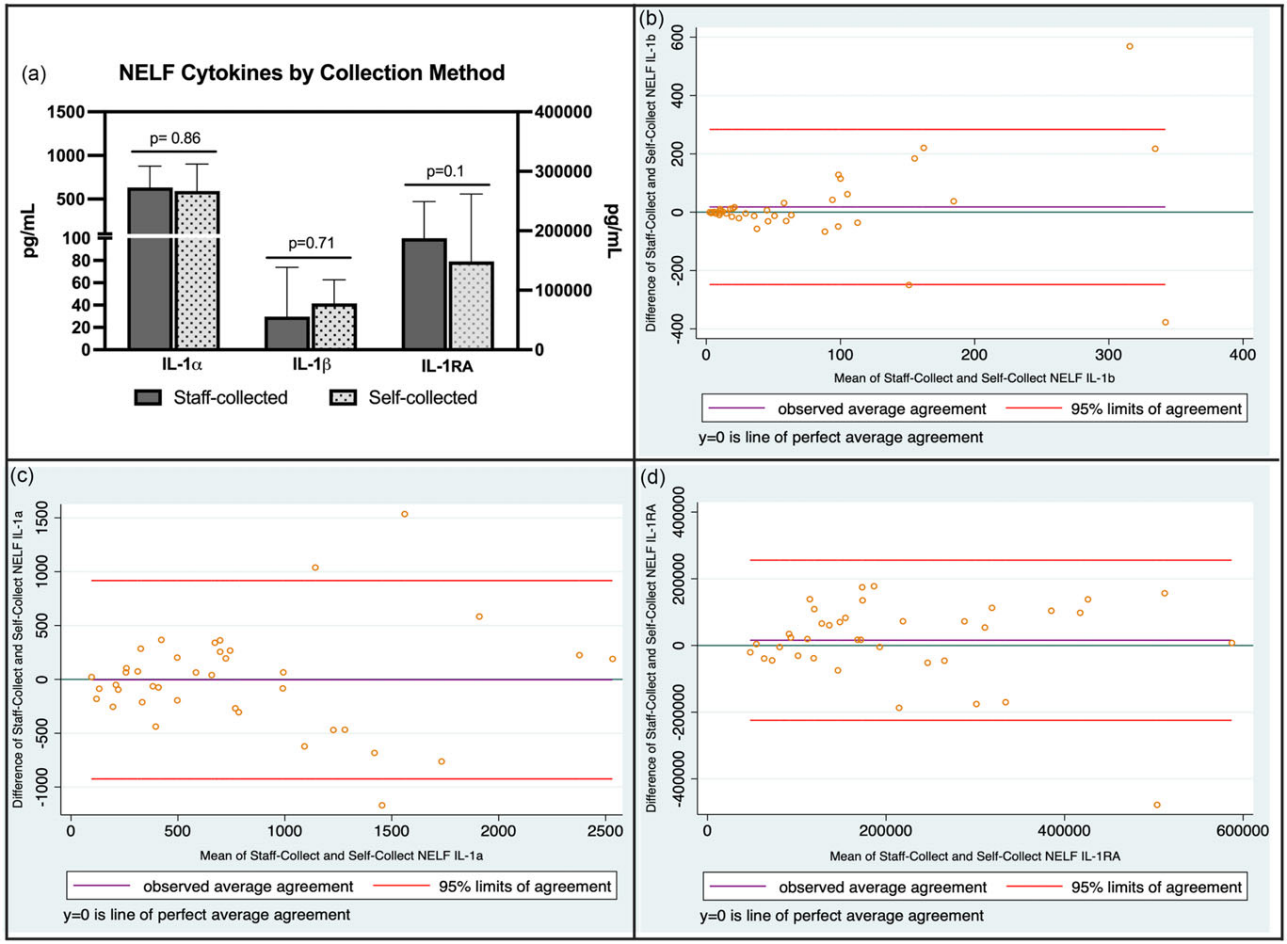
(a) Baseline cytokine concentrations in NELF samples under different collection and storage conditions (median (95% CI) concentrations). Samples were compared using Wilcoxon matched pairs signed rank test with significance level set at *p* < 0.05. (b–d) Bland Altman plots of nasal cytokines collected under the two conditions.

Only two sets of self-collected samples experienced significant temperature excursions during the shipping process. Both sets of samples were delayed in reaching the lab because the carrier was experiencing shipping delays and were excluded from the analysis. We compared the values of cytokines of interest (IL-1α, IL-1β, IL-1RA) from NELF samples collected during the initial in-person study visit to samples self-collected at home by the participant, usually within 1–2 days of the initial study visit. Cytokine data were available for 39 of the 40 participants; one participant did not provide a self-collected baseline NELF sample. Ninety percent (35/39) of home baseline samples were collected within one day of the in-person study visit. NELF concentrations of IL-1α, IL-1β, and IL-1RA from home self-collected NELF samples were not statistically significantly different from samples collected during the in-person study visit and immediately frozen at −80 C (Figure 4a). Bland Altman plots were generated to assess agreement between the two sampling methods (Figure 4b). For IL-1β, there was little bias, indicating good agreement overall between the two methods, with a few outliers at higher averages. For IL-1α and IL-1RA, more bias was observed and wider limits of agreement, indicating reduced agreement between methods, particularly at high cytokine concentrations.

### Technical challenges

During the virtual monthly follow up visits, the majority of the visit was spent troubleshooting problems with the devices. The most common problems encountered were difficulty connecting and syncing the spirometer and digital inhaler to the participant’s smartphone. This occurred for several reasons: difficulty connecting to WiFi, insufficient battery charge on the devices, Bluetooth was unknowingly turned off, and use of outdated versions of the app. As described above, we encountered inappropriate alerts being sent to participants to collect nasal samples when the exacerbation criteria were not met, most often due to software erroneously indicating a ≥20% fall in FEV1, either due to poor spirometry technique that was not detected by the software, or for unknown reasons when the actual decline in FEV1 was <20%. The majority of these errors occurred in the first few months of conducting the study. We utilized a customized version of the commercially available app, which required some refinement during the early portion of our study. After the problems with the app were resolved, we had very few erroneous alerts for the remainder of the study. Study coordinators frequently performed re-coaching on spirometry technique, which was challenging to do in a virtual encounter. In three instances, participants met criteria for asthma exacerbation, but the app did not send a sample collection alert to the participant or study coordinator. There were six instances in which exacerbation criteria were met but due to delays in communication between the participants and study coordinator, nasal samples were not collected.

## Discussion

This observational study was designed to allow for nearly all study visits, data collection, and biological sample collection to be completed remotely during the height of the SARS-CoV-2 pandemic. The pandemic dramatically changed medical care and clinical research, limiting in-person visits and restricting aerosolizing procedures like spirometry. At the same time, it accelerated the development of infrastructure for virtual and remote care. Although the pandemic has ended, virtual care remains the preferred way to receive care and participate in clinical research activities for many individuals. Our study population of adolescents and young adults was racially diverse with a significant degree of asthma morbidity, reflected in the high proportion of participants with ≥2 exacerbations in the past year, prescription of biologic therapies, and history of hospitalization for status asthmaticus, including ICU admissions. Despite normal average lung function and ACT scores, 78% of participants experienced one or more exacerbations during the 12-month study period. Exacerbations occurred in two peaks, one in the spring (April–May) and a second in the fall/early winter (November–December). The probable trigger for each exacerbation was not recorded but given that our young study population was highly atopic (see Table 1), this was most likely related to seasonal allergen exposure (spring peak) and viral respiratory infection (fall/early winter peak). These findings underscore the need for longitudinal clinical data collection in asthma, a condition marked by temporal variability in symptoms and airway obstruction.

Nearly three-quarters of enrolled participants completed the entire 12-month study. The hybrid study design, which allows for mostly remote participation, enables patients to participate in clinical research by removing the burden of frequent in-person visits requiring missed school or workdays. This approach increases access to clinical research participation to the populations most vulnerable and most burdened by poor asthma control. Historically, clinical research designs have excluded participants unable to attend frequent in-person visits and who were not able to be strictly adherent to daily study procedures and data collection. As a result, study populations often fail to reflect the broader asthma patient population, excluding many individuals who could have benefited from participation and contributed valuable insights into the generalizability of study outcomes. We acknowledge that the 27% rate of attrition is higher than some high impact clinical trials. However, these types of trials usually enroll highly motivated individuals with less severe disease and lower racial and socioeconomic diversity. In contrast, we feel that our racially, ethnically and socioeconomically diverse study sample better reflects the population at greatest risk of short-term and long-term morbidity from asthma, the individuals that stand to benefit most from treatment but who are usually excluded from research.

Adherence to daily spirometry measurement and survey completion in our study was variable but lower than desired, with participants completing spirometry and symptom surveys about one-half and one-third of enrolled days, respectively. This finding aligns with our observations in a previous study of home spirometry use in adolescents that did not include additional incentives for good adherence [23]. These observations suggest that monetary incentives do not substantially influence adherence behavior in adolescents with asthma, a finding that has been replicated in other studies [24,25]. The digital rescue inhalers demonstrated that over 90% of participants utilized rescue albuterol during the study period. Digital inhalers have the advantage of passive data collection and allow for more accurate reporting of rescue medication use compared to self-report. Of note, many of the participants in our study also had access to nebulized albuterol prescribed by their clinician, which was not monitored electronically. Therefore, it is possible that reliance on digital inhalers could underestimate the use of rescue medication.

Even with these limitations, our study yielded robust data, capturing 121 true exacerbation events (105 detected through software alerts, and 16 that were self-reported or identified through EHR) and resulting collection of 65 sets of NELF samples (54% of events). While this collection rate appears low, 28% of events occurred too soon with respect to the previous exacerbation prompting study staff to instruct participants not to collect samples. We required a sufficient wash-out period between exacerbations to ensure that what was measured reflected a new discrete exacerbation event and not carryover from the previous exacerbation. The high rate of these events reflects the burden of disease in our sample rather than an inherent flaw in the design. The remaining events did not have corresponding samples collected for reasons such as delays in communication between study staff and participants, participant refusal to collect samples, and lack of alerts generated to prompt collection. Most collected samples were received by the lab without significant temperature excursions. Comparison of measured analytes between samples collected at the in-person study visit then immediately frozen and the courier-delivered frozen samples shipped by participants showed no statistically significant differences. Tests of agreement between methods demonstrated good agreement between sampling techniques for IL-1β, with reduced agreement for IL-1α and IL-1RA. The modest number of observations in this small sample of asthmatics likely influenced some of the observed lack of agreement. We cautiously interpret these findings as being supportive of a self-collection approach to obtaining biological samples for analysis of IL-1β concentrations in the nasal mucosa. However, we acknowledge that these observations are applicable to this specific type of sampling method and are not necessarily generalizable to other collection methods or types of biological samples.

The study team faced several challenges during the study period. Technical difficulties with the smartphone app and devices were frequent. The most common problems included Bluetooth connectivity issues between the devices, app crashing, insufficient battery charge, difficulty resetting passwords, and inappropriate sample collection alerts. Inappropriate alerts to collect samples were triggered by application errors, falsely low FEV1 measurement from poor technique, or unknown reasons. We attribute most of these early errors to the initial implementation of the customized smartphone application, as the system required refinement during the first few months of the study. Notably, error rates declined substantially after this initial adjustment period. Only one instance of inappropriate NELF sample collection occurred. Combined with the overall low risk of harm using this sampling technique, the safety profile of our protocol was favorable. Several participants required re-training on proper home spirometer technique. There were also instances in which exacerbation criteria were met but collection alerts were not pushed out by the app to the participant. When collection alerts were received by the study team, a research coordinator reviewed their data and contacted the participant to guide them on whether to collect NELF samples. It is possible that this approach resulted in higher frequencies of sample collection than if participants were only prompted to collect by the app collection alerts, a limitation of our study. There were also instances of delays in communication between the research coordinator and the participant following a collection alert that resulted in samples not being collected. Combined with the lower than desired adherence to daily study procedures, this suggests that decentralized study designs depend heavily on an effective clinical research team and regular, frequent communication with study participants. A list of potential advantages and disadvantages of decentralized studies with remote data collection is found in Table 2.

**Table 2.** Advantages and disadvantages of decentralized research and remote data collection

PRO	CON
Increased accessibility of clinical trial participation, aiding in participant recruitment, sample diversity, and generalizability of trial results	Frequent troubleshooting of equipment and connectivity issues
Convenience for participants, promoting study retention	Requirement for trained staff with technical expertise
Data collected better reflects day-to-day habits and behaviors compared to recall data collected at traditional study visits	Need for frequent re-training of participants on equipment use
Allows for robust data collection and longitudinal assessment rather than a single point in time	Time consuming monitoring of data collection to ensure accuracy by study staff
	Additional cost of equipment and monitoring
	Potential for inaccurate or missed alerts
	Potential for missing data due to low adherence to study procedures

In conclusion, decentralized study designs with near-fully remote visits and data collection should be further studied as a means of conducting clinical research in asthma. These models support broader representation by reducing traditional barriers to research participation, such as transportation challenges, missed work or school, and geographic limitations. However, this approach also presents challenges and limitations, including the time and technical expertise required of study staff, the risk of low adherence to daily study procedures, the need for accurate and reliable software programs for data tracking, quality control of biological samples, and frequent and proactive communication between the study team and participants.

To fully realize the potential of decentralized trials, future efforts should focus on optimizing these designs to ensure they are both accessible and scientifically rigorous. This includes investing in accurate, reliable and user-friendly digital tools, culturally responsive engagement strategies, and flexible protocols that accommodate a wide range of participant needs and circumstances. By addressing these factors in study planning and budgeting, decentralized designs can significantly improve the quality and generalizability of clinical research by including populations historically underrepresented in asthma research.
